# Investigation of a Progressive Relaxation Training Intervention on Precompetition Anxiety and Sports Performance Among Collegiate Student Athletes

**DOI:** 10.3389/fpsyg.2020.617541

**Published:** 2021-03-17

**Authors:** Dongmei Liang, Shuqing Chen, Wenting Zhang, Kai Xu, Yuting Li, Donghao Li, Huiying Cheng, Junwei Xiao, Liyi Wan, Chengyi Liu

**Affiliations:** ^1^School of Physical Education & Sports Science, South China Normal University, Guangzhou, China; ^2^National Sports Science Experimental Teaching Demonstration Center, South China Normal University, Guangzhou, China

**Keywords:** progressive relaxation training, precompetition anxiety, self-confidence, sports performance, collegiate student athletes

## Abstract

This study aims to investigate whether simple and convenient progressive relaxation training (PRT) is effective in enhancing collegiate student athletes’ mental health and sports performance. An experimental group of 14 (6 female) and a control group of 10 (5 female) collegiate student athletes were recruited from among track and field athletes who were preparing for provincial competition. The experimental group was exposed to a PRT intervention in 30-min sessions conducted twice per week for a duration of one month. At baseline, the Competitive State Anxiety Inventory-2 (CSAI-2), State-Trait Anxiety Inventory (STAI), and Eysenck Personality Questionnaire-Revised Short Scale for Chinese (EPQ-RSC) were completed, while only the CSAI-2 was reassessed at one, two, and three weeks after initiation of the intervention. Additionally, within half a day after completing all one’s individual competition events, the CSAI-2 was again assessed in the two groups recalling their memory of their precompetition state anxiety. Then, the differences in the three dimensions of the CSAI-2 between the two groups at the five time points introduced above were compared. This study also explored whether PRT affected sports performance, defined by the athletes reaching their best records or not, by logistic regressive analysis. This study found significant between-group differences in the self-confidence dimension score at the second and third time points. Through logistic regression analysis, a positive effect of PRT was found for the enhancement of sports performance. In sum, PRT showed positive effects on precompetition state self-confidence and enhanced sports performance among collegiate student athletes.

## Introduction

Mental health (especially depression and anxiety) has been increasingly emphasized among athletes (Reardon et al., 2019; Rice et al., 2019, 2016). A meta-analysis examining anxiety among elite athletes reported that approximately 75% of the included references had been published in the previous five years (Rice et al., 2019). Drew et al. (2018) investigated five aspects of illness symptoms, including deteriorated mental health status and high-pressure conditions, in 317 athletes participating in 11 sports three months before the 2016 summer Olympic Games; their findings revealed that anxiety and depression status were the main factors resulting in illness, as all participants reported at least one symptom in the month before the investigation. In addition to the emphasis of anxiety in regard to the mental health of athletes, continuing discussions are being conducted on its relationship with sports performance (Kleine et al., 1988; Kleine, 1990; Hardy and Hagtvet, 1996; Englert and Bertrams, 2012; Rathschlag and Memmert, 2015; Steinberg and Doppelmayr, 2015; Siart et al., 2017; Yang et al., 2019).

Competitive anxiety is one kind of state anxiety and is also called competitive state anxiety (Gould et al., 1987; Craft et al., 2003; Tsopani et al., 2011). Competitive state anxiety exists in different phases of a competition (including precompetition). Given its important impact on sports performance, investigations have focused on precompetition anxiety (Gould et al., 1987; Jokela and Hanin, 1999; Fu, 2000; Woodman and Hardy, 2003; Tsopani et al., 2011; De Pero et al., 2013; Zenebe et al., 2016; Munoz et al., 2017; Souza et al., 2019). Among the published reports, the Competitive State Anxiety Inventory-2 (CSAI-2) (Martens et al., 1990) was the most cited instrument and reflects the effects of state anxiety on sports performance from multiple dimensions including somatic anxiety, cognitive anxiety, and self-confidence (Gould et al., 1987; Jokela and Hanin, 1999; Craft et al., 2003; Woodman and Hardy, 2003; De Pero et al., 2013; Zenebe et al., 2016; Munoz et al., 2017; Souza et al., 2019). Since the translation of the CSAI-2 and its introduction to China (Zhu, 1994), the 27-item Chinese version of the CSAI-2 has been continuously used (Fu, 2000; Liu et al., 2015), including its recent application among track and field collegiate student athletes (Xu et al., 2017; Fang, 2019), reflecting the credibility and validity of the Chinese version of the CSAI-2 in evaluating precompetition anxiety.

In addition to state anxiety, sports performance can also be impacted by trait anxiety (Horikawa and Yagi, 2012; Judge et al., 2016). The State-Trait Anxiety Inventory (STAI) (Spielberger et al., 1983) is a valid instrument for evaluating anxiety (Julian, 2011) and has also been applied in the evaluation of competition anxiety (Turner and Raglin, 1996; Yang et al., 2007; Silva et al., 2012). Reports have shown no difference between the CSAI-2 and STAI in evaluating precompetition anxiety traced back from five days after the competition (Wilson et al., 2000). Differences in personality, especially differences in the neuroticism dimension, also induce different effects on the experience and the level of deterioration due to anxiety (Lahey, 2009; Balyan et al., 2016; Petito et al., 2016; Dal et al., 2018; Rocha and Osório, 2018).

Determining how to decrease anxiety and enhance sports performance has always been emphasized (Scott-Hamilton et al., 2016; Bühlmayer et al., 2017; Beatty and Janelle, 2019; Chen et al., 2019). Studies have examined how classic psychological skill training methods (such as relaxation, mental imagery, and self-talking) change psychological status and enhance skill performance and how new applications can be carried out. For example, Hagan et al. (2017) found that long-term positive precompetition psychological status was expected among athletes when routine psychological skill training was added in a continuous training program. Kuan et al. (2018) concluded that mental image training combined with relaxation music enhanced delicate skill performance. Additionally, significant effects of relaxation training for decreasing anxiety have been repeatedly found (Manzoni et al., 2008; Rumbold et al., 2012; Kim and Kim, 2018).

Unlike elite athletes, collegiate student athletes face the same academic and social pressure as their peer schoolmates in addition to the need to enhance personal sports skills and acquire better competitive achievements. However, although collegiate student athletes are faced with significantly less competitive pressure than elite athletes, due to the lack of skilled experience coping with different environmental stresses, valid consultation and services should be available to them in case of impaired functioning induced by various pressures. Therefore, the importance of mental health interventions should not be neglected among these individuals (Shannon et al., 2019). Recently, a consensus regarding the process of recognition and referral of collegiate student athletes with psychological concerns has been reached among different associations of the States (Neal et al., 2013). Additionally, randomized controlled trials among collegiate student athletes examining the effects of asking for aid in sustaining mental health, based on a particular model, were conducted (Eisenberg, 2014). Similarly, in China, collegiate athletic teams are always short of professional psychological consultants; therefore, easy and convenient interventions to improve mental health as well as enhance sports performance are practical solutions.

Progressive relaxation training (PRT), also called Jacobson’s progressive relaxation or progressive muscle relaxation (PMR), is one kind of relaxation training as well as a coping strategy applied in systematic desensitization (Wolpe, 1958) of cognitive behavioral therapy (CBT). It is a standard instrument used by therapists in CBT or behavioral therapy. Focusing on self-motion combined with repeated rotations through “contract–relaxation–recontract” cycles of muscle movements, it may be that athletes have a sophisticated understanding of voluntary relaxation of their whole-body muscle, better cognize intense reflection, and thus control the intensity of the whole-body muscle, leading to peace and decreased negative emotions such as anxiety, depression, and fear (Zhao et al., 2016). Therefore, through peace and relaxation of the whole-body muscle, PRT inhibits anxiety induced by particular environments, such as general anxiety reactions (Dolbier and Rush, 2012; Allison et al., 2019; Quinones and Griffiths, 2019), specialized speech phobia (Hazlett-Stevens and Borkovec, 2001), and preexamination anxiety (Wachelka and Katz, 1999; Dehghan-Nayeri and Adib-Hajbaghery, 2011; O’Donnell and Dunlap, 2019; Gallego-Gómez et al., 2020), as reported in recent research. Among these studies, the most commonly reported effects pertained to the release of anxiety among collegiate students (Wachelka and Katz, 1999; Dehghan-Nayeri and Adib-Hajbaghery, 2011; Dolbier and Rush, 2012; Allison et al., 2019). Through changing anxiety status, PRT can efficiently enhance personal performance (Wachelka and Katz, 1999; Kim, 2008; Gallego-Gómez et al., 2020). Meanwhile, PRT has been shown to be capable of decreasing anxiety and pressure, as well as promoting better performance among professional athletes (Keilani et al., 2016). However, only a study of decreasing physiological stress reactivity among collegiate student athletes is reported (Hunt et al., 2018).

Therefore, there are reports of decreased anxiety and enhanced performance among collegiate students through PRT. A report has also demonstrated the effect on decreasing physiological stress reactivity among collegiate student athletes by PRT. However, there is no report of the application of PRT for decreasing state anxiety or enhancing sports performance among collegiate student athletes as far as we know. In this study, investigation of the inhibiting effect of PRT on precompetition anxiety was first considered. Meanwhile, a longitudinal analysis of changes in trends of precompetition anxiety under different conditions was provided. Additionally, whether the intervention was good for athletes in achieving their best personal performance was explored. The CSAI-2 and STAI were assessed for the evaluation of precompetition anxiety. Considering the higher probability of anxious experiences in those with higher neuroticism personality scores and their impact on precompetition anxiety, the Eysenck Personality Questionnaire-Revised Short Scale for Chinese version (EPQ-RSC) was also assessed to test the personality of participants. There were two hypotheses: first, PRT is capable of reducing precompetition anxiety among collegiate student athletes; second, PRT is beneficial in enhancing sports performances of collegiate student athletes.

## Materials and Methods

### Participants

This study recruited 25 participants from a collegiate track and field team who were preparing for provincial college-student sports games. All participants were randomly divided into the experimental group and control group. At the baseline, there were 14 subjects in the experimental group (male:female, 8:6) and 11 subjects in the control group (5:6). Although one female participant in the control group completed all tests, she knew that she would not participate in the competition. Compared with other participants in the control group and all participants in the experimental group, there was a lower probability for this participant to experience a precompetition anxiety state; therefore, her data were not included, and the number (and gender ratio) in the control group was 10 (5:5). All subjects gave their informed consent for inclusion before they participated in the study. The study was conducted in accordance with the Declaration of Helsinki, and the protocol was approved by the Human Research Ethics Committee of South China Normal University.

### Research Procedure

In this study, the recruited participants were first randomly assigned to the experimental group and the control group based on gender. Then, after twice intervention, we noticed that two participants in the experimental group were not willing to join the PRT intervention while four in the control group were interested to know what was going on with their peers after their training (the PRT training is conducted posttraining each time). Thus, we replaced the unwilling two participants with the four interested initial ‘controls’. The baseline test started after the decision of 14 subjects in the experimental group and 10 subjects in the control group, and the instruments included the CSAI-2, STAI, and EPQ-RSC. After completing the tests, all participants began the 1-month precompetition preparatory training for six days a week, with one group resting on Saturday and the other on Sunday, or the coach adjusted the relaxation and training contents according to training conditions of athletes (the timing was uncertain under this condition). In the first week of training, participants of the two groups took part in the same preparation process together. After a high-intensity training day (twice per week), the participants in the experimental group joined a 30-min PRT session, which included communication before the intervention, the 15-min intervention, and feedback after the intervention. After the PRT session, the participants arranged their own activities as usual. The participants in the control group arranged their own activities after each training session as usual. All subjects in the experimental group and the control group maintained their original way of life and academic study and engaged in the same precompetition training. The only difference was that subjects in the experimental group received PRT twice a week.

After one week of preparatory training (and the intervention in the experimental group), the CSAI-2 was completed for the participants in the two groups. In the second week, all arrangements, including way of life, academic study, training, and intervention (for the experimental group), were the same as in the first week. After two weeks of preparatory training/intervention (for the experimental group), the CSAI-2 was completed again in the two groups of participants. In the third week, the two groups of participants maintained the same arrangements as the first and second weeks. After three weeks of preparatory training/intervention, the CSAI-2 was completed again in the two groups of participants, and in the fourth week of preparatory training, the two groups of participants maintained the same arrangements as those in the first to third weeks.

**FIGURE 1:**
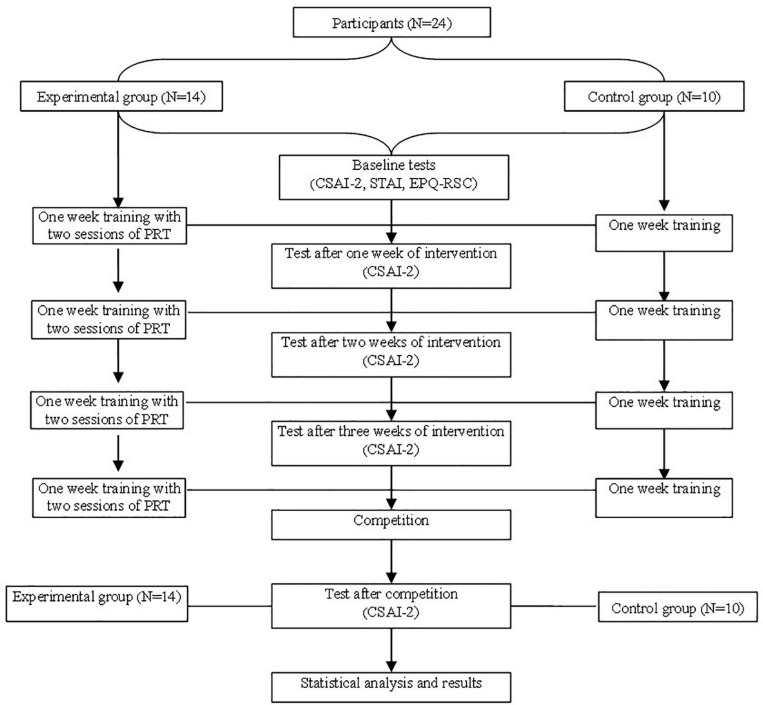
Research procedure.

After four weeks of preparatory training/intervention, the two groups of participants took part in the competition together, and the coach recorded the results of all participants in the preliminary and final round of each event. Each subject was tested with the CSAI-2 again within half a day (3–10 hrs) after completing all their individual competition events to report their memory of their precompetition state anxiety. Meanwhile, information including each participant’s most and less skilful events, the best results of them, and the experience in both events was collected. The research procedure is shown in Figure 1.

### Measurement Instruments

#### Competitive State Anxiety Inventory-2 (CSAI-2)

In this study, the form E version of the CSAI-2 was used, which was adapted from the original form A. It includes three subscales, including cognitive state anxiety, somatic state anxiety, and state self-confidence, with nine questions in each subscale and 27 questions in total on the scale. The score of all subscales can range from 9 to 36, and a higher score means higher cognitive state anxiety, somatic state anxiety, or state self-confidence. All questions scored from 1 to 4 reflected enhanced intensity with the exception of question 14, which was reverse scored. In the norm of the Chinese version of the CSAI-2 (Zhu, 1994), the internal consistency of the three subscales ranged from 0.68 to 0.72. As the subscales of cognitive state anxiety and somatic state anxiety showed a low positive relationship (*r* = 0.11–0.14), while state self-confidence showed a low negative relationship (*r* = −0.12 to −0.21) to scores on factor Q4 and factor O in Cattell’s 14 FP, the questionnaire demonstrates higher concurrent validity with the relevant state anxiety scale and trait anxiety scale. The medium positive correlations (*r* = 0.49–0.62) between cognitive state anxiety and somatic state anxiety show good independence of each subscale. In addition, the average correlation coefficients between scores on the cognitive and somatic state anxiety subscales and sports performances in the Chinese athletes sample were 0.2 to 0.26, and the corresponding correlation coefficient of the scores on the state confidence subscale was −0.32, which reflects a higher efficacy of the CSAI-2 questionnaire in predicting sports performance.

#### State-Trait Anxiety Inventory (STAI)

This study used the Chinese version of the STAI, which consists of guidelines and two subscales including 40 descriptive questions in total. Questions 1 to 20 represent the state anxiety inventory (S-AI), whereas questions 21 to 40 represent the trait anxiety inventory (T-AI). Each question on the STAI was evaluated from 1 to 4: on the S-AI, 1 represents not at all, 2 is somewhat, 3 is a medium degree, and 4 is very obvious; on the T-AI, 1 is almost nothing, 2 is somehow, 3 is often, and 4 is almost always. Then, the cumulative scores on the S-AI and T-AI were calculated with a minimum value of 20 and a maximum value of 80, reflecting the degree of state or trait anxiety, respectively. It has been reported that the test–retest reliability of the T-AI was 0.73 to 0.86, and that of the S-AI was 0.16 to 0.62. The correlation coefficients for the scores on the S-AI and T-AI were 0.77 to 0.84 with satisfactory structural validity (Wang et al., 1999).

#### Eysenck Personality Questionnaire-Revised Short Scale for Chinese (EPQ-RSC)

The EPQ-RSC used in this study was revised from the Eysenck Personality Questionnaire Brief Scale (EPQ-RS) by Qian et al. (2000). It consisted of four subscales, including extraversion (E), neuroticism (N), psychoticism (P), and validity (L) subscales, with 12 items on each subscale. The test–retest reliability of the scale was reported to be 0.67 to 0.88 after three weeks; the split-half reliability was 0.51 to 0.77, and the internal consistency was 0.54 to 0.78. The correlations between the original scale and the Chinese version of the EPQ were 0.27 to 0.64 (only the P subscale was 0.27, less than 0.5), which shows good validity.

### Introduction and Implementation of Progressive Relaxation Training

#### Introduction of Progressive Relaxation Training

Progressive relaxation training is also called Jacobson’s progressive relaxation (Jacobson, 1938). It consists of two parts: posture preparation and 20 practices. The 20 practices begin with breathing, followed by twice relaxation of all the individual parts of the upper limbs in sequence. The relaxation of each body part includes four steps: first, contraction of the part; second, a pause for 5 s to experience the feeling of contraction; third, relaxation of the part; and fourth, a pause for 5 s to experience the feeling of relaxation. The relaxation sequence of all parts is as follows: first, the upper limbs; second, the head; third, the trunk; and fourth, the lower limbs. The relaxation of each following part also includes the four steps. Finally, the relaxation of the combined parts of the upper limbs occurs first, and then all parts of the lower limbs are relaxed together to complete the whole training process.

#### Implementation of Progressive Relaxation Training

First, for the posture preparation, participants need to loosen tight clothes and decorations that would hinder practice and then sit on a sofa or chair, with the two arms and hands being placed flat on the armrests of sofa or chair, the two legs naturally stretched forward, and the head and upper body gently leaning on the back of sofa; during this process, the participants need to sit as comfortably as possible to improve and enhance the effects of the practices.

Second, the 20 practices start with breathing, followed by upper limb relaxation, including the left hand, right hand, left biceps, right biceps, and fists; relaxation of the head, including the forehead, eyes, chins, neck, and lips; and relaxation of the trunk, including the shoulders and belly. The relaxation steps for each part are introduced in section “Introduction of Progressive Relaxation Training”. Relaxation of the upper limbs, head, and trunk is followed by relaxation of the lower limbs with the sequence of toes, heel, calf, and thigh of the left leg, the right leg, and then both legs. The relaxation steps for each part are the same as those introduced in section “Introduction of Progressive Relaxation Training”. Finally, relaxation of the upper limbs, head, and trunk is combined, and then relaxation of all parts of the head, trunk, and lower limbs is combined to complete the whole relaxation process.

### Definition and Classification of Sports Performance

To evaluate the performance of the athletes, a criterion of reaching their best record was set. The two groups of athletes were classified based on their performance as best-record athletes versus non–best-record athletes. A detailed description is introduced below.

The definition was based on the events in which the athletes were most skilful/less skilful, the best records of the athletes in these most skilful/less skilful events, and the participated events and best records in these participated events. If the records in the participated events were the same or better than the personal best record for their most skilful/less skilful events, they were classified as best-record athletes. First, for athletes who only participated in their most skilful or less skilful events (nine athletes in two groups), classification was based on the records from these particular most skilful or less skilful events, and there were two athletes categorized as best-record athletes. Second, for athletes participating in both their most skilful and less skilful events (twelve athletes in two groups), classification was based on their best records from the participated events, and seven athletes were categorized as best-record athletes. Third, athletes who participated in events that were neither of their most skilful nor of their less skilful events (three athletes in two groups) were categorized as non–best-record athletes, as their recent results could not be compared to previous records. In total, nine athletes were categorized as best-record athletes, including seven athletes from the experimental group and two athletes from the control group.

### Definition of Training Experience

Regarding the definition of training experience (years), with the exception of one athlete with no training experience in her less skilful event, all other athletes had training experiences with both their most skilful and less skilful events (23 athletes). For the one athlete, her training experience with the most skilful event was accepted as her training experience. For the other athletes, there were two kinds of definitions. For athletes with the same training experiences at their most skilful and less skilful items (12 athletes), their training experiences were defined as their experience with these events. For athletes with different training experiences between their most skilful and less skilful events (11 athletes), according to the introduction in section “Definition and Classification of Sports Performance,” their training experiences were defined based on three conditions. First, for individuals who participated in only a single event (i.e., their most skilful or less skilful event), their training experiences in this event were defined as their individual training experience (four athletes). Second, for athletes who participated in both their most skilful and less skilful events, with the exception of two athletes whose training experiences with their most skilful events were shorter than their less skilful events, this duration was accepted as their training experiences. For other athletes, all of their training experiences were defined as their training experiences with their most skilful events (three athletes). Third, for athletes who participated in items being neither their most skilful nor least skilful event, their training experiences were defined by the longest experience among the events (two athletes).

## Statistical Analysis

In the data preprocessing, missing data were first checked in both groups and replaced by the mean value of the group. Then, a test of normality by skewness and kurtosis with a *p*-level of 0.05 was conducted on all demographic data, including age, training experience (years), height, body mass index (BMI), and scores of all measurement instruments, including the EPQ-RSC, STAI, and CSAI-2, at each time point. Next, outliers were processed first by the results of the boxplot if the value was more than 1.5 standard deviations (SDs) from the mean; second, suspected outliers were confirmed by the Dixon Q test (Dixon test or Q test) (Dixon, 1953) (if the data were normally distributed) or the Peirce method (Peirce, 1852) (if the data were not normally distributed). For the Q test, the steps were as follows. First, values were arranged in a sequence from high to low; second, the Q value of the data for the group was calculated according to the formula:

$$\text{Q}=\frac{/\mathrm{Outliers}-\mathrm{Proximity}\mathrm{value}/}{/\mathrm{Maximum}-\mathrm{Minimum}/}$$

Third, referring to the Q table, the cut-off Q value was obtained using two indexes, namely, the number of participants in the group and the confidence level (such as 90%, 98%, and 99%), to judge whether a suspected outlier was accepted as an outlier by comparing the differences between the calculated and the cut-off Q values. If the calculated Q value was less than the cut-off Q value from the Q table (this study set a 99% confidence level with a *p*-level of 0.005), the suspect data point was classified as a normal value; if not, the suspect data point was classified as an outlier. For the Peirce method, the steps were as follows. First, the mean and SD of the group with the suspected outliers were calculated. Second, referring to Peirce’s table for R (Ross, 2003), the cut-off R value was obtained through two indexes, namely, the group sample size and the number of suspected outliers. Third, the maximum allowable deviation (MAD = |*x*_*i*_ – *x*_*m*_|_*max*_ = SD * *R*) was calculated. Fourth, the actual deviation (Devact = |*x*_*i*_ – *x*_*m*_|) was calculated, which was the absolute value of the difference between the suspected outlier and the mean of its group. Finally, the two deviations were compared. The suspected outliers were classified as normal values if Devact was less than MAD; if not, they were classified as outliers. Through the Peirce method, one outlier from the training experience data set and one outlier from the cognitive anxiety of the CSAI-2 data set at baseline in the experimental group were confirmed as outliers and respectively replaced by the means of the group.

To compare group differences at baseline, Fisher’s exact tests and independent *t* tests were applied. Because of the insufficient sample size (the expected frequency of women in the control group was less than 5), Fisher’s exact test was applied to compare the differences in gender distribution between two groups. The differences in age, BMI, training experience, EPQ-RSC subscale and total scores, and STAI subscale and total scores between the two groups were compared through independent *t* tests, which were also applied to compare the differences in CSAI-2 scores between the two groups at baseline and at the time points one, two, three, and four weeks after the initiation of the intervention. Because the sample size was less than 20, Hedges’ *g* was used to calculate the effect sizes. Wang et al. (2010) introduced different ranges of Hedges’ *g* in relation to clinical effects: 0–0.19: negligible effect, 0.20–0.49: small effect, 0.50–0.79: moderate effect, ≥ 0.80: large effect. Hedges’ *g* was calculated as follows:

$${\mathrm{Hedges}}^{\prime }g=\frac{M_{1}\text{-}M_{2}}{S_{\text{pooled}}}$$

As there were no significant differences between the two groups in demographic data, including gender, age, BMI, training experience, and factors that might impact state anxiety, including EPQ total and subscale scores and STAI total and subscale scores, repeated-measures ANOVA without covariates was applied to detect changes at each time point for each dimension of the CSAI-2, the changes in each dimension score of the CSAI-2 over time, the differences between two groups, and the interactive effect of time and group. Fisher’s exact tests and independent *t* tests were carried out in SPSS 21.0, and repeated-measures ANOVA was processed in GraphPad Prism 8.0.

To examine whether the PRT intervention had an effect on sports performance, the results of whether individuals reached their best records were taken as the dependent variables, while participation in the intervention was taken as the fixed independent variable to form logistic regression models to investigate the predictive effect of PRT on sports performance. Logistic regression analysis was processed in GraphPad Prism 8.0.

## Results

### Basic Information of Participants

Fisher’s exact test showed that there was no significant difference in gender distribution between the two groups, *p >* 0.05.

Independent tests showed that there were no significant differences between the two groups in age, *t* = −0.276, *p* = 0.785, Hedges’ *g* = 0, 95% CI [−1.307, 1.000]; BMI, *t* = 0.117, *p* = 0.909, Hedges’ *g* = 0.1, 95% CI [−1.552, 1.726]; training experience, *t* = 0.311, *p* = 0.758, Hedges’ *g* = 0.13, 95% CI [−1.491, 2.018]; EPQ-RSC P subscale, *t* = −0.775, *p* = 0.446, Hedges’ *g* = 0, 95% CI [−1.89, 0.861]; E subscale, *t* = 0.059, *p* = 0.953, Hedges’ *g* = 0, 95% CI [−1.943, 2.057]; N subscale, *t* = −0.73, *p* = 0.473, Hedges’ *g* = 0.3, 95% CI [−3.676, 1.762]; L subscale, *t* = 1.04, *p* = 0.31, Hedges’ *g* = 0.4, 95% CI [−1.107, 3.336]; the total scores of the EPQ-RSC, *t* = −0.067, *p* = 0.947, Hedges’ *g* = 0.03, 95% CI [−3.212, 3.012]; S-AI, *t* = −0.571, *p* = 0.574, Hedges’ *g* = 0.24, 95% CI [−13.965, 7.936]; T-AI, *t* = −0.822, *p* = 0.42, Hedges’ *g* = 0.34, 95% CI [−12.889, 5.575]; STAI-T, *t* = −0.702, *p* = 0.49, Hedges’ *g* = 0.29, 95% CI [−26.393, 13.050]; CSAI-2 somatic state anxiety, *t* = 0.025, *p* = 0.981, Hedges’ *g* = 0.01, 95% CI [−4.777, 4.891]; cognitive state anxiety, *t* = 1.229, *p* = 0.232, Hedges’ *g* = 0.50, 95% CI [−1.812, 7.082]; state self-confidence, *t* = 1.585, *p* = 0.127, Hedges’ *g* = 0.64, 95% CI [−0.912, 6.826].

The basic information of the participants and the differences at baseline between the two groups on the EPQ-RSC, STAI, and CSAI-2 subscales are shown in Table 1.

**TABLE 1:** Basic information of participants (Baseline, *M ± SD*).

Group	Gender (M:F)	Age	BMI (kg/m^2^)	Training experience	P	E	N	L	EPQ-RSC-T	SAI	TAI	STAI-T	SA^#^	CA^#^	SC^#^
Experimental (*n* = 14)	8:6	20.85 ± 1.46	21.04 ± 0.92	4.31 ± 2.23	2.29 ± 1.27	8.36 ± 2.02	4.14 ± 3.06	5.21 ± 2.72	20 ± 3.88	46.79 ± 12.48	46.14 ± 9.99	92.93 ± 21.72	21.36 ± 6.01	23.24 ± 3.70	22.86 ± 4.15
Control (*n* = 10)	5:5	21 ± 1.16	20.96 ± 2.23	4.05 ± 1.74	2.8 ± 1.99	8.3 ± 2.71	5.1 ± 3.32	4.1 ± 2.38	20.1 ± 3.21	49.8 ± 13.14	49.8 ± 11.76	99.6 ± 24.66	21.3 ± 5.03	20.6 ± 6.77	19.9 ± 4.98
*T-*value	–	−0.276	0.117^†^	0.311	−0.775	0.059	−0.73	1.04	−0.067	−0.571	−0.822	−0.702	0.025	1.229	1.585
*P*-value	1.000^&^	0.785	0.909^†^	0.758	0.446	0.953	0.473	0.31	0.947	0.574	0.42	0.49	0.981	0.232	0.127

*M±SD, mean ± standard deviation; M:F,male:female; BMI, body mass index; P, psychoticism; E, extraversion; N, neuroticism; L, validity scale; SAI, state anxiety inventory; TAI, trait anxiety inventory; STAI-T, State-Trait Anxiety Inventory. ^&^Fisher’s exact test (two-sided). ^†^Value of *T* and *P* (two-sided test) with unequal variance in the Levene test. ^#^Subscale scores of CSAI-2 for two groups at baseline.*

### Comparison of CSAI-2 Subscale Scores Between the Two Groups at Each Time Point

#### Comparison of Somatic Anxiety at Each Time Point

**FIGURE 2:**
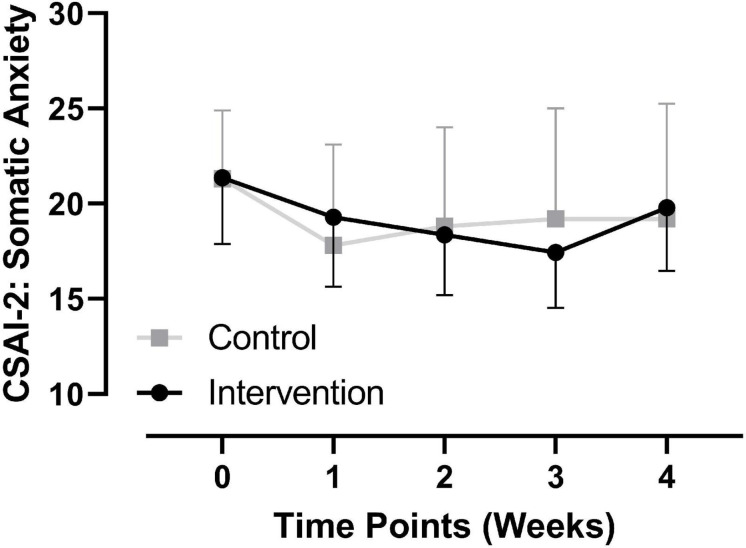
Comparison of somatic anxiety at each time point between groups.

Regarding somatic anxiety, repeated-measures ANOVA showed that the main effects of time, group, and the interactive effect of time × group were not significant, *p >* 0.05 (see Figure 2). Regarding between-group comparisons at different time points, there were no significant differences at baseline (see section “Basic Information of Participants”); 1 week, *t* = 0.529, *p* = 0.602, Hedges’ *g* = 0.22, 95% CI [−4.339, 7.311]; 2 weeks, *t* = −0.17, *p* = 0.866, Hedges’ *g* = 0.07, 95% CI [−5.833, 4.947]; 3 weeks, *t* = −0.661, *p* = 0.516, Hedges’ *g* = 0.28, 95% CI [−7.333, 3.790], and 4 weeks, *t* = 0.203, *p* = 0.841, Hedges’ *g* = 0.09, 95% CI [−5.408, 6.579] after the initiation of the intervention (see Table 2 and Figure 2).

**TABLE 2:** Differences in CSAI-2 subscale scores at each time point between the two groups (*M ± SD*).

Group	1 week			2 weeks			3 weeks			4 weeks		
	SA	CA	SC	SA	CA	SC	SA	CA	SC	SA	CA	SC
Experimental (*n* = 14)	19.29 ± 6.32	19.71 ± 6.13	22.79 ± 3.89	18.36 ± 5.49	20 ± 5.90	24.71 ± 2.76	17.43 ± 5.03	18.21 ± 6.77	23.14 ± 4.61	19.79 ± 5.74	19 ± 6.01	22.93 ± 5.44
Control (*n* = 10)	17.8 ± 7.41	22.4 ± 7.53	19 ± 4.94	18.8 ± 7.27	21.2 ± 7.51	19.9 ± 5.59	19.2 ± 8.12	21.1 ± 7.71	19.8 ± 5.96	19.2 ± 8.46	20.9 ± 7.61	19.5 ± 4.53
*T*-value	0.529	−0.962	2.102	−0.17	−0.439	2.515^†^	−0.661	−0.972	1.552	0.203	−0.684	1.628
*P-*value	0.602	0.346	**0.047**^∗∗^	0.866	0.665	**0.027**^†**^	0.516	0.342	0.135	0.841	0.501	0.118

M ± SD, mean ± standard deviation; SA, somatic anxiety; CA, cognitive anxiety; SC, self-confidence. ^†^The value of *T* and *P* (two-sided test) with unequal variance in the Levene test. **p < 0.05.

#### Comparison of Cognitive Anxiety at Each Time Point

**FIGURE 3:**
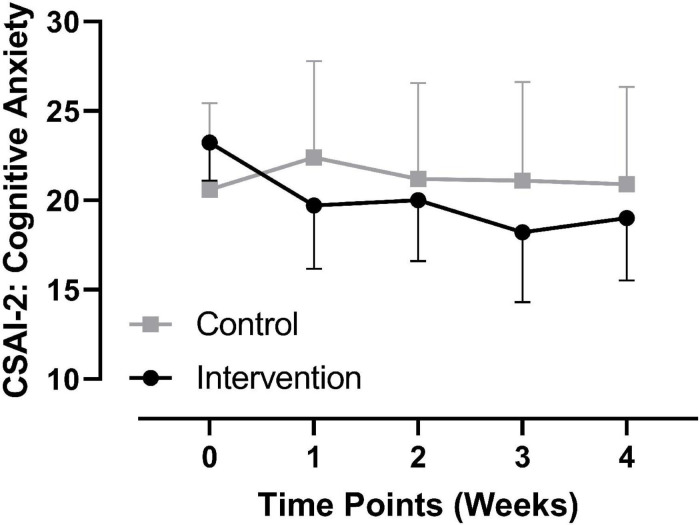
Comparison of cognitive anxiety at each time point between groups.

Regarding cognitive anxiety, repeated-measures ANOVA showed that the main effects of time, group, and the interactive effect of time × group were not significant,*p >* 0.05 (see Figure 3). Regarding between-group comparisons at different time points, there were no significant differences at baseline (see section “Basic Information of Participants”); 1 week, *t* = −0.962, *p* = 0.346, Hedges’ *g* = 0.4, 95% CI [−8.473, 3.101]; 2 weeks, *t* = −0.439, *p* = 0.665, Hedges’ *g* = 0.18, 95% CI [−6.871, 4.471]; 3 weeks, *t* = −0.972, *p* = 0.342, Hedges’ *g* = 0.4, 95% CI [−9.043, 3.272], and 4 weeks, *t* = −0.684, *p* = 0.501, Hedges’ *g* = 0.29, 95% CI [−7.663, 3.863] after the initiation of the intervention (see Table 2 and Figure 3).

#### Comparison of State Self-Confidence at Each Time Point

**FIGURE 4:**
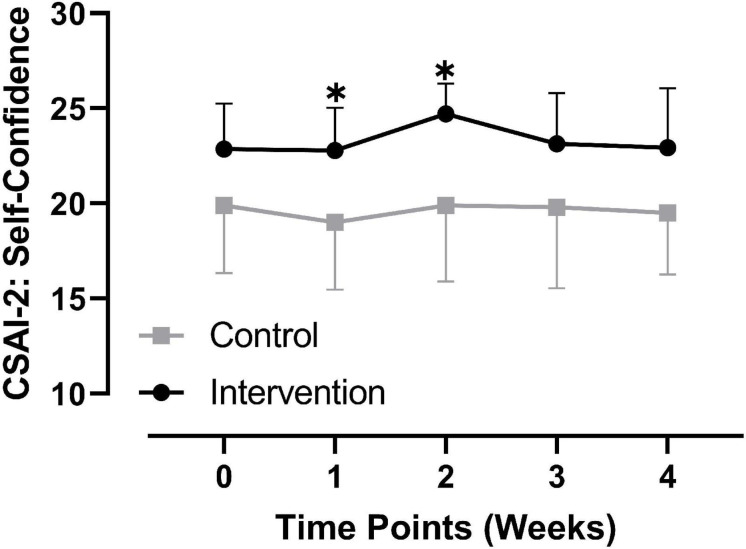
Comparison of self-confidence at each time point between groups.

Regarding state self-confidence, repeated-measures ANOVA showed that the main effects of time and the interactive effect of time × group were not significant,*p >* 0.05 (see Figure 4). The main effect of group was significant, *F*(1,22) = 6.134, *p* = 0.021. Regarding between-group comparisons at each time point, there were no significant differences at baseline (see section “Basic Information of Participants”); 3 weeks, *t* = 1.552, *p* = 0.135, Hedges’ *g* = 0.6, 95% CI [−1.124, 7.810]; 4 weeks, *t* = 1.628, *p* = 0.118, Hedges’ *g* = 0.65, 95% CI [−0.940, 7.797] after the intervention. There were significant differences at 1 week, *t* = 2.102, *p* = 0.047, Hedges’ *g* = 0.8, 95% CI [0.050, 7.521] and 2 weeks, *t* = 2.515, *p* = 0.027, Hedges’ *g* = 1, 95% CI [0.649, 8.979] after initiation of the intervention (see Table 2 and Figure 4).

### Effect of PRT on Sports Performance—Logistic Regression Analysis

#### Inclusion of Variables

The participants were categorized into two groups based on the criterion of reaching one’s best record or not. Demographic factors and subscale and total scores on the EPQ-RSC and STAI were compared between groups. Before the comparison was made, normality of the distributions was tested for all indexes (except gender) in the two groups. Meanwhile, suspected outliers were detected. For suspected outliers in the group with data that were normally distributed, Dixon’s Q test (Dixon, 1953) was applied to define the outliers. If the data were not normally distributed, Peirce’s method (Peirce, 1852) was applied. One outlier among the BMI data was defined in the best-record athlete group; one outlier among the P subscores and one among the E subscores on the EPQ-RSC were defined in the other group, and all were replaced by the means of their groups. Normality of the distribution was again tested, and all indexes for the two groups were normally distributed.

Independent *t* tests were applied for the group difference comparison of all indexes (except gender), and Fisher’s exact test was applied in the group difference comparison of gender (see Table 3 for all results). According to the suggestion of Hosmer, Lemeshow, and Sturdivant (Hosmer et al., 2013), *p* < 0.20 (0.25) was taken as the criterion of the *t* test, BMI (*X*_2_) was then included, together with “intervention (*X*_1_)” as variables for the multiple logistic regression analysis to predict whether the participants will reach their best records in the competition. Meanwhile, in consideration of the possibility of an interaction effect between BMI and intervention, BMI × intervention (*X*_3_) was included in the regression model; therefore, a model with three factors was set as Model 1: *Y*_1_ = *B*_0_ + *B*_1_*X*_1_ + *B*_2_*X*_2_ + *B*_3_*X*_3_. Excluding the interaction effect, the logistic regression model was set as Model 2: *Y*_2_ = *B*_0_ + *B*_1_*X*_1_ + *B*_2_*X*_2_. With the subsequent exclusion of the effect of BMI (*X*_2_), a more concise model was set as Model 3: *Y*_3_ = *B*_0_ + *B*_1_*X*_1_.

**TABLE 3:** Index comparisons between groups defined by reaching best record (*M ± SD*).

	Gender		BMI	Training								
Group	(M:F)	Age	(kg/m^2^)	experience	P	E	N	L	EPQ-RSC-T	SAI	TAI	STAI-T
Reached (*n* = 9)	5:4	21.11 ± 1.45	21.58 ± 0.60	4.50 ± 3.84	2.44 ± 1.33	8.22 ± 2.54	4.11 ± 2.67	5.44 ± 1.74	20.22 ± 3.49	47.89 ± 7.29	47.11 ± 7.46	95.00 ± 13.54
Not reached (*n* = 15)	8:7	20.79 ± 1.26	20.87 ± 1.72	4.57 ± 1.86	2.24 ± 1.27	8.76 ± 1.62	4.80 ± 3.45	4.33 ± 2.97	19.93 ± 3.69	48.13 ± 15.13	48.00 ± 12.46	96.13 ± 27.25
*T*-value	–	0.570	1.465^†^	−0.057	0.384	−0.638	−0.513	1.018	0.189	−0.045	−0.193	−0.116
*P*-value	1.000*	0.574	0.159^†^	0.955	0.705	0.530	0.613	0.320	0.852	0.964	0.848	0.909

M ± SD, mean ± standard deviation; M:F,male:female; BMI, body mass index; P, psychoticism; E, extraversion; N, neuroticism; L, validity subscale; EPQ-RSC-T, total score on the Eysenck Personality Questionnaire-Revised Short Scale for Chinese; SAI, state anxiety inventory; TAI, trait anxiety inventory; STAI-T, State-Trait Anxiety Inventory. *Fisher’s exact test (two-sided). ^†^Value of T and P (two-sided test) with unequal variance in the Levene test.

### Model Building and Comparative Analysis of Different Models

Different notation was taken to indicate whether one’s best record was reached (“1” for “yes” and “0” for “no”). Based on the data from the two groups for all variables (*X*_1_, *X*_2_, *X*_3_), three logistic regression models were built, and the likelihood ratio test (LRT) was applied to evaluate the goodness-of-fit between the models and data. The parameters for each model are listed in Table 4. Three models were built as *Y*_1_ = −4.46 - 31.19*X*_1_ + 0.14*X*_2_ + 1.55*X*_3_, *Y*_2_ = −14.8 + 1.88*X*_1_ + 0.61*X*_2_, and *Y*_3_ = −1.39 + 1.39*X*_1_. The criterion for a significant test of the parameters in the three models was set as a one-sided *p* < 0.10 (0.05) as a significant (very significant) difference (Table 4). Three aspects were considered in the criterion definition: first, the two groups all contained small samples; second, this study was a pilot study to investigate the direction/sign of the effect in applying PRT among collegiate students; and third, to avoid type III errors, it was inferred that the direction of an effect was the same as the observed direction.

**TABLE 4:** Logistic regression models of PRT in predicting sports performance.

Model	Variable	Coeff.	Std.Err.	*z*	*p*^§^	95% CI
1	Intervention	–31.19	20.95	–1.49	0.068*	[−80.58, 6.59]
	BMI	0.14	0.43	0.33	0.371	[−0.70, 1.15]
	Intervention * BMI	1.55	0.99	1.57	0.058*	[−0.21, 3.88]
	Constant	–4.46	9.38	–0.48	0.316	[−26.94, 13.19]
2	Intervention	1.88	1.16	1.62	0.053*	[−0.13, 4.62]
	BMI	0.61	0.42	1.45	0.074*	[−0.13, 1.58]
	Constant	–14.80	9.52	–1.55	0.061*	[−36.81, 1.52]
3	Intervention	1.39	0.95	1.45	0.074*	[−0.37, 3.51]
	Constant	–1.39	0.79	–1.75	0.040**	[−3.28, 0]

Coeff, estimated coefficient; Std. Err., standard error; Z, Coeff/Std. Err. ^§^One-sided values. *p < 0.10. ^∗∗^*p* < 0.05.

As the parameter to evaluate goodness-of-fit for the models, area under the receiver operating characteristic curve (AUC) was linked to the discriminative ability of the models to the predicted results. According to the suggestion of Hosmer et al. (2013), 0.8 ≤ AUC < 0.9 represents excellent discrimination; 0.7 < AUC < 0.8 represents acceptable discrimination; the AUCs of Model 1 and Model 2 discriminated whether the participants reached their best records, Model 1: AUC = 0.82, SE = 0.09, 95% CI [0.65, 0.99], *P* = 0.009; Model 2: AUC = 0.77, SE = 0.10, 95% CI [0.57, 0.97], *P* = 0.03. However, Hosmer et al. (2013) consider 0.5 < AUC < 0.7 as poor discrimination, while Model 3: AUC = 0.66, SE = 0.12, 95% CI [0.43, 0.88], *P* = 0.21. Therefore, we made comparisons between Model 1 and Model 3 as well as between Model 2 and Model 3. It indicated that Model 3 was the better model to fit the data. In the two comparisons, as the three models are nested models, the LRT was applied to define the better model. The LRT statistic = [Deviance (simpler model) - Deviance (more complex model)], which indicates how much smaller the deviance is for the more complex model. The value of this statistic is used to calculate a *p*-value. A small *p*-value (*p <* 0.05) suggests rejecting the null hypothesis, which indicates that the simpler model is correct. In the comparison between Model 2 and Model 3, the LRT statistic was 2.51 (*p* = 0.11, >0.05); in the comparison between Model 1 and Model 3, the LRT statistic was 5.44 (*p* = 0.07, >0.05), all indicate the acceptance of the simpler model. Therefore, the more concise Model 3 was better than Model 1 and Model 2.

#### Analysis of the Effects of the Intervention on Sports Performance

**FIGURE 5:**
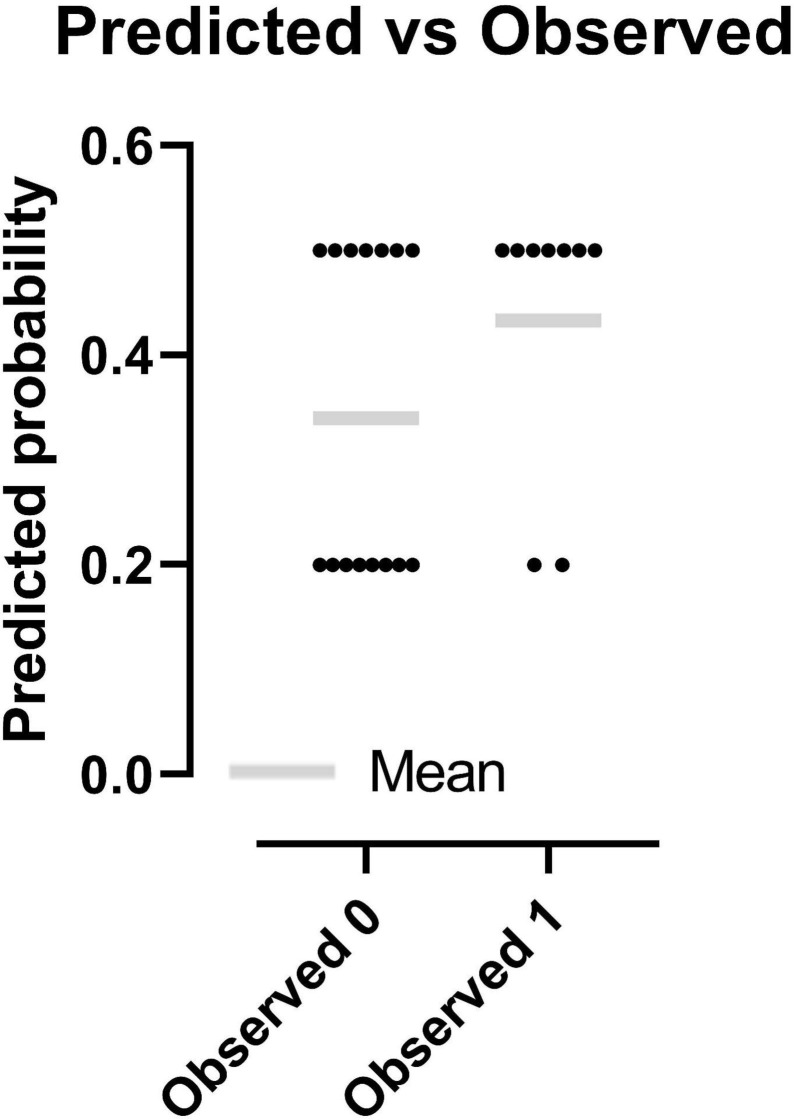
Comparsion of predicted probability between classifications.

Analysis of Model 3 indicated the following. First, it fits best with the data. Second, the “predicted-observed” figure (Figure 5) based on it indicates that the predictive results were different for the participants with or without PRT intervention. Combined with the results of the classification table (Table 5), which was matched with the “predicted-observed” figure, it shows that, for the participants without PRT intervention, the predictive ratios of whether they would or would not reach their best records in the competition were nearly equivalent (47.67%:53.33%); however, for the participants with the PRT intervention, the predictive ratio of whether they would reach their best records in the competition was 77.78%. In summary, Model 3 demonstrated the “right” predictive ratio of sports performance at 62.5%. To conclude, Model 3 fits best with the data, showing a predictive ratio of 77.78% that the participants with the PRT intervention would reach their best records in the competition. The results from the logistic regression analysis reflect the positive effect of PRT on sports performance.

**TABLE 5:** Classification Table of “Predicted-Observed” Results from Model 3.

Classification	Predicted 0	Predicted 1	Total	Correctly classified (%)
Observed 0	8	7	15	53.33
Observed 1	2	7	9	77.78
Total	10	14	24	62.50

## Discussion

In this study, PRT with strong maneuverability, convenience, and ease of implementation was applied to intervene in the precompetition anxiety of track and field collegiate student athletes. Through a week-by-week intervention during the one month preparatory period before the competition with measurements at one time point before the intervention, three time points during the intervention, and one time point after the competition (with results tracing back to precompetition), we compared the differences in the scores on the state anxiety subscales of the CSAI-2 between the experimental and control groups. Significant group differences in state self-confidence one and two weeks after initiation of the PRT intervention were found. Through logistical regression analysis, PRT showed a positive effect on the performance of collegiate track and field athletes. This study is not only an innovative demonstration of the effects of PRT as an intervention for precompetition anxiety and enhanced sports performance but also provides longitudinal changes in precompetition anxiety under different conditions. Practically, this study is an initial exploration of an effective, simple, and easy-to-conduct measurement that enhances the mental health and sports performance of collegiate student athletes.

Individual factors have significant effects on anxiety. When considering their related effects, personality is considered first. Both three- and five-factor personality models include neurotic traits, which are closely related to mental health, physical health, and quality of life (Lahey, 2009); these traits affect the cognitive and physiological responses of athletes during competition (Balyan et al., 2016) and predict negative mental health results together with serotonin transporter gene polymorphism and competition pressure (Petito et al., 2016). When applied in the discussion of specific events, Dal et al. (2018) reported that neuroticism regulates the prediction of somatic anxiety on the activity of the sympathetic nervous system before and during archery competition. Through applying the three-factor EPQ-RSC, this study found that there was no significant difference between the two groups in different dimensions (including neuroticism) of personality, which provided a basis for the follow-up study examining the effect of the intervention. In addition to personality factors, trait anxiety is another important factor affecting the generation of anxiety as well as sports performance. Individuals with higher trait anxiety were more likely to experience increased state anxiety under stressful conditions, which in turn affected their target performance (Horikawa and Yagi, 2012). As trait anxiety impairs sports performance, applying an intervention before or during competition will be beneficial and result in good performance (Judge et al., 2016). In this study, there were no significant differences in trait anxiety scores measured across dimensions of the STAI between the two groups at baseline, which also laid a foundation for the follow-up examination of the effects of the intervention.

From the comparison of each dimension of the CSAI-2 at all time points, state self-confidence showed a significant between-group difference at one and two weeks after initiation of the intervention (*p* < 0.05). There were no significant between-group differences at any time point in the other two dimensions. Therefore, we suggest that PRT may have a positive effect on the self-confidence of collegiate track and field athletes. However, this effect did not continue through the end of the intervention. As shown in Figure 4, self-confidence in the experimental group showed a rapid decline from two to three weeks after initiation of the intervention, while it remained constant over the same time period in the control group. Moreover, comparing baseline values with those from three weeks after initiation of the intervention, there was only a slight increase (mean value from 22.86 to 23.14) in self-confidence in the experimental group, while these values remained unchanged (both 20.09) in the control group. Since three weeks after initiation of the intervention was in the late phase of the preparation as there was only one week left for the competition, and although somatic anxiety and cognitive anxiety in the experimental group at this phase continued to decline (refer to Figures 2, 3, respectively), the athletes continued to face high levels of competition pressure. For young collegiate athletes with poorer performances and a relative lack of competitive experience, although the PRT intervention continued to be applied, their state self-confidence did not continuously increase or remain unchanged. However, as self-confidence in the experimental group at three weeks after initiation of the intervention was still slightly higher than the baseline, combined with the changing trend in the previous three time points, the trend of PRT in decreasing precompetition anxiety through enhancing self-confidence among collegiate athletes was clear.

Along with this trend of PRT on self-confidence, this study also found that PRT showed a positive effect on sports performance defined by reaching one’s best records, suggesting that PRT may improve sports performance of collegiate track and field athletes. Consistent with Keilani et al. (2016), who found that different psychological techniques can reduce depressive symptoms in professional athletes and improve their psychological skills and the ability to cope with stress, psychological techniques (such as PRT) are effective in enhancing sports performance. We have discussed the positive effects of PRT on the self-confidence aspect of state anxiety. Although this study did not investigate the effect of changes in state anxiety (self-confidence) on performance, many supportive findings have been provided by previous studies. For example, Mabweazara et al. (2016) found that precompetition state anxiety of high school swimming athletes significantly predicted their performance. Craft et al. (2003) reported that the self-confidence aspect of state anxiety evaluated by the CSAI-2 showed the strongest and most consistent relationship with performance, while the relationship between other dimensions and performance was weak. Tsopani et al. (2011) found that state self-confidence was the only factor that significantly predicted performance among rhythmic athletes, suggesting that strategies with the effect of improving self-confidence should be adopted to enhance performance. Modroño and Guillen (2011) found that athletes with better overall rankings demonstrated lower somatic anxiety, and the top five athletes showed higher self-confidence, suggesting that athletes with better performance may have lower anxiety and higher self-confidence. A study with women volleyball players found that there was a significant correlation between state self-confidence and performance, and athletes with higher self-confidence may show better performance (Zenebe et al., 2016). Research with elite female track and field athletes found that high levels of self-confidence helped in reducing precompetition cognitive anxiety and somatic anxiety, and state self-confidence was the key factor in achieving excellent results (Munoz et al., 2017). Therefore, further investigations are suggested to explore the role of changes in state self-confidence as a contributor to the effect of PRT on sports performance.

Although there were no significant between-group differences in somatic and cognitive anxiety at each time point, the changing trend of the two dimensions at each time point between the two groups was obviously the opposite. For somatic anxiety, the changing trend from one to three weeks after initiation of the intervention between the two groups was obviously opposite. The experimental group showed a continuous downward trend from baseline to three weeks after initiation of the intervention, and the lowest score at three weeks after initiation of the intervention was lower by nearly 3 SDs (from the baseline mean of 21.36, SD of 6.01, to a mean of 17.43, SD of 5.03 at three weeks after initiation of the intervention), while the scores in the control group went from 20.36 to 19.36 over the same period. Regarding cognitive anxiety, the changing trend from baseline to three weeks after initiation of the intervention between the two groups was also different. The experimental group changed from 23.24 to 18.21, representing a difference of more than 2 SDs. The control group showed a slight increase from 19.91 to 21.45. Therefore, although there was no significant between-group difference at each time point of the intervention in somatic and cognitive anxiety, the positive effect of PRT on the two measures was also obvious. Further comparisons of the changing trends in the two groups for these two dimensions from the end of the intervention to 1 hr before the competition time point showed an opposite changing trend; for the experimental group, there was an upward trend, while the control group remained unchanged. Thus, the potential benefits of PRT in decreasing somatic and cognitive state anxiety of collegiate student athletes are highly suggested.

Overall, regarding the changes in the three dimensions of the CSAI-2 from baseline (time point “0” in Figures 2–4) to the last time point of the intervention (time point “3” in Figures 2–4), the changing trends in the two groups were different. In the experimental group, somatic anxiety continuously decreased, whereas cognitive anxiety decreased gradually. State self-confidence showed a gradual increasing trend at all time points with the exception of the final time point during the intervention, which showed a decreasing trend. In the control group, somatic anxiety showed a rapid decrease and then continued to increase, cognitive anxiety showed a slow increase and then decreased, and state self-confidence showed a slow decrease and then a trend of recovering and maintaining. From the end of the intervention to the precompetition time point, the somatic anxiety of the experimental group showed a trend of a rapid increase, while the cognitive anxiety showed a slow increase, and the state self-confidence remained unchanged. The somatic and cognitive anxiety of the control group remained unchanged, whereas state self-confidence slightly declined. Overall, with the exception of the change from baseline to one week after initiation of the intervention, the changing trend in the two groups for somatic anxiety was opposite, and the changing trend in the two groups for state self-confidence was only the same from one to two weeks after initiation of the intervention, but opposite for all other time points and all time points of cognitive anxiety. Therefore, this study also provided a detailed description of the longitudinal changes over time (Badami et al., 2012) for the different dimensions of precompetition state anxiety not only in the undisturbed control state but also in the PRT intervention state, which provides a vivid picture for collegiate athletes, coaches, and administrators to consult in their management of athletic training, mental health, and sports performance.

Results from this study are suggested to be with limited application in the psychological intervention among professional teams. As the research question was from the training practice of collegiate student athletes, and all participants are undergraduate students at the major of physical education. Also, as an initial investigation, small, unequal sample size and unparalleled gender were applied, which might be factors affecting the results. Therefore, caution should be taken when thinking of applying the conclusions, even among collegiate student athletes. On the contrary, no significant results concluded in this study do not mean their invalidity in the athletic training practice.

Regarding the lack of sports medicine services, as well as professional psychological consultants in collegiate sports teams, this study investigated whether easy and convenient PRT is an effective method to intervene in precompetition anxiety and enhance sports performance. PRT was found to have a positive effect on self-confidence and sports performance improvements; meanwhile, its potential benefits in decreasing somatic and cognitive state anxiety of collegiate student athletes were highly suggested. In summary, this study provides a reference for collegiate athletes, coaches, and administrators to consult in their management of athletic training, mental health, and sports performance.

## Data Availability Statement

The raw data supporting the conclusions of this article will be made available by the authors, without undue reservation.

## Ethics Statement

The studies involving human participants were reviewed and approved by Human Research Ethics Committee of South China Normal University. All participants provided their written informed consent to participate in this study.

## Author Contributions

DmL designed the experiment, directed data collection process, did data analysis and wrote the manuscript. SC coordinated the experiment under the supervision of DmL. WZ collected data, aided in data analysis and participated in the manuscript preparation. KX conducted the intervention and tracked participants. YL collected references and participated in the manuscript preparation. DhL aided in reference collection and summary as well as participated in the manuscript preparation. HC, JX, and LW aided in the manuscript preparation. CL contributed in revising the manuscript critically. All authors read and approved the submitted version.

## Conflict of Interest

The authors declare that the research was conducted in the absence of any commercial or financial relationships that could be construed as a potential conflict of interest.

## References

[B1] Allison S. Hamilton K. I. Yuan Y. Hague G. W. (2019). Assessment of progressive muscle relaxation (pmr) as a stress-reducing technique for first-year veterinary students. *J. Vet. Med. Educ.* 47 737–744. 10.3138/jvme.2018-0013 31738679

[B2] Badami R. VaezMousavi M. Wulf G. Namazizadeh M. (2012). Feedback about more accurate versus less accurate trials: differential effects on self-confidence and activation. *Res. Q. Exerc. Sport* 83 196–203. 10.1080/02701367.2012.10599850 22808705

[B3] Balyan K. Y. Tok S. Tatar A. Binboga E. Balyan M. (2016). The relationship among personality, cognitive anxiety, somatic anxiety, physiological arousal, and performance in male athletes. *J. Clin. Sport Psychol.* 10 48–58. 10.1123/jcsp.2015-0013

[B4] Beatty G. F. Janelle C. M. (2019). Emotion regulation and motor performance: an integrated review and proposal of the temporal influence model of emotion regulation (TIMER). *Int. Rev. Sport Exerc. Psychol.* 13 266–296. 10.1080/1750984X.2019.1695140

[B5] Bühlmayer L. Birrer D. Röthlin P. Faude O. Donath L. (2017). Effects of mindfulness practice on performance-relevant parameters and performance outcomes in sports: a meta-analytical review. *Sports Med.* 47 2309–2321. 10.1007/s40279-017-0752-9 28664327

[B6] Chen J. H. Tsai P. H. Lin Y. C. Chen C. K. Chen C. Y. (2019). Mindfulness training enhances flow state and mental health among baseball players in Taiwan. *Psychol. Res. Behav. Manag.* 12 15–21. 10.2147/PRBM.S188734 30613170 PMC6307497

[B7] Craft L. L. Magyar T. M. Becker B. J. Feltz D. L. (2003). The relationship between the competitive state anxiety inventory-2 and sport performance:A meta-analysis. *J. Sport Exerc. Psychol.* 25 44–65. 10.1123/jsep.25.1.44

[B8] Dal N. Tok S. Dogan E. Balikçi I. Zekioglu A. Çatikkas F. (2018). Somatic anxiety may represent archers’ actual autonomic nervous system activity but how: moderating role of personality traits. *Univers. J. Educ. Res.* 6 1831–1836. 10.13189/ujer.2018.060828

[B9] De Pero R. Minganti C. Pesce C. Capranica L. Piacentini M. F. (2013). The relationships between pre-competition anxiety, self-efficacy,and fear of injury in elite teamgym athletes. *Kinesiology* 45 63–72.

[B10] Dehghan-Nayeri N. Adib-Hajbaghery M. (2011). Effects of progressive relaxation on anxiety and quality of life in female students: a non-randomized controlled trial. *Complement. Ther. Med.* 19 194–200. 10.1016/j.ctim.2011.06.002 21827933

[B11] Dixon W. J. (1953). Processing data for outliers. *J. Biom.* 9 74–89. 10.2307/3001634

[B12] Dolbier C. L. Rush T. E. (2012). Efficacy of abbreviated progressive muscle relaxation in a high-stress college sample. *Int. J. Stress Manag.* 19 48–68. 10.1037/a0027326

[B13] Drew M. Vlahovich N. Hughes D. Appaneal R. Burke L. M. Lundy B. (2018). Prevalence of illness, poor mental health and sleep quality and low energy availability prior to the 2016 summer olympic games. *Br. J. Sports Med.* 52 47–53. 10.1136/bjsports-2017-098208 29056598

[B14] Eisenberg D. (2014). *Developing and Evaluating a Model Program for Supporting the Mental Health of Student Athletes.* Available online at: https://www.ncaa.org/about/resources/research/developing-and-evaluating-model-program-supporting-mental-health-student-athletes

[B15] Englert C. Bertrams A. (2012). Anxiety, ego depletion, and sports performance. *J. Sport Exerc. Psychol.* 34 580–599. 10.1123/jsep.34.5.580 23027229

[B16] Fang S. L. (2019). Relationship between pre-competition anxiety and coping strategies of college athletes in track events. *Zhejiang Sport Sci.* 41 81–88.

[B17] Fu M. Q. (2000). A study of intensity, frequency & direction of pre-competition anxiety of sportsmen at differenct levels. *Int. J. Psychol.* 35 56–56.

[B18] Gallego-Gómez J. I. Balanza S. Leal-Llopis J. García-Méndez J. A. Oliva-Pérez J. Doménech-Tortosa J. (2020). Effectiveness of music therapy and progressive muscle relaxation in reducing stress before exams and improving academic performance in Nursing students: a randomized trial. *Nurse Educ. Today* 84:104217. 10.1016/j.nedt.2019.104217 31683132

[B19] Gould D. Petlichkoff L. Simons J. Vevera M. (1987). Relationship between competitive state anxiety inventory-2 subscale scores and pistol shooting performance. *J. Sport Exerc. Psychol.* 9 33–42. 10.1123/jsp.9.1.33

[B20] Hagan J. E. Jr. Pollmann D. Schack T. (2017). Elite athletes’ In-event competitive anxiety responses and psychological skills usage under differing conditions. *Front. Psychol.* 8:2280. 10.3389/fpsyg.2017.02280 29312103 PMC5743917

[B21] Hardy L. Hagtvet K. A. (1996). Anxiety and performance:measurement and modelling issues. *Anxiety Stress Coping* 9 R5–R5.

[B22] Hazlett-Stevens H. Borkovec T. D. (2001). Effects of worry and progressive relaxation on the reduction of fear in speech phobia: an investigation of situational exposure. *Behav. Ther.* 32:503. 10.1016/S0005-7894(01)80033-2

[B23] Horikawa M. Yagi A. (2012). The relationships among trait anxiety, state anxiety and the goal performance of penalty shoot-out by university soccer players. *PLoS One* 7:e35727. 10.1371/journal.pone.0035727 22539998 PMC3335041

[B24] Hosmer D. W. Jr. Lemeshow S. Sturdivant R. X. (2013). *Applied Logistic Regression.* New York, NY: JohnWiley & Sons.

[B25] Hunt M. Rushton J. Shenberger E. Murayama S. (2018). Positive effects of diaphragmatic breathing on physiological stress reactivity in varsity athletes. *J. Clin. Sport Psychol.* 12 27–38. 10.1123/jcsp.2016-0041

[B26] Jacobson E. (1938). *Progressive Relaxation.* Chicago, IL: University of Chicago Press.

[B27] Jokela M. Hanin Y. L. (1999). Does the individual zones of optimal functioning model discriminate between successful and less successful athletes? A meta-analysis. *J. Sports Sci.* 17 873–887. 10.1080/026404199365434 10585167

[B28] Judge L. W. Urbina L. J. Hoover D. L. Craig B. W. Judge L. M. Leitzelar B. M. (2016). The impact of competitive trait anxiety on collegiate powerlifting performance. *J. Strength Cond. Res.* 30 2399–2405. 10.1519/JSC.0000000000001363 26881803

[B29] Julian L. J. (2011). Measure of anxiety. *Arthritis Care Res.* 63 S467–S472. 10.1002/acr.20561 22588767 PMC3879951

[B30] Keilani M. Hasenöhrl T. Gartner I. Krall C. Fürnhammer J. Cenik F. (2016). Use of mental techniques for competition and recovery in professional athletes. *Wien. Klin. Wochenschr.* 128 315–319. 10.1007/s00508-016-0969-x 26932798 PMC4875065

[B31] Kim H. S. Kim E. J. (2018). Effects of relaxation therapy on anxiety disorders: a systematic review and meta-analysis. *Arch. Psychiatr. Nurs.* 32 278–284. 10.1016/j.apnu.2017.11.015 29579524

[B32] Kim Y. (2008). The effect of improvisation-assisted desensitization, and music-assisted progressive muscle relaxation and imagery on reducing pianists’ music performance anxiety. *J. Music Ther.* 45 165–191. 10.1093/jmt/45.2.165 18563972

[B33] Kleine D. (1990). Anxiety and sport performance: a meta-analysis. *Anxiety Res.* 2 113–131. 10.1080/08917779008249330

[B34] Kleine D. Sampedro R. M. Melo S. L. (1988). Anxiety and performance in runners: effects of stress and anxiety on physical performance, Anxiety Research. *Anxiety Res.* 1 235–246. 10.1080/08917778808248722

[B35] Kuan G. Morris T. Kueh Y. C. Terry P. C. (2018). Effects of relaxing and arousing music during imagery training on dart-throwing performance, physiological arousal indices, and competitive state anxiety. *Front. Psychol.* 9:14. 10.3389/fpsyg.2018.00014 29459837 PMC5807418

[B36] Lahey B. B. (2009). Public health significance of neuroticism. *Am. Psychol.* 64 241–256. 10.1037/a0015309 19449983 PMC2792076

[B37] Liu J. X. Lin L. Zhang Y. Li K. X. Qiu W. H. (2015). The document metrological review of sports psychology research since the 1980s in China. *China Sport Sci.* 35 74–82.

[B38] Mabweazara S. Leach L. Andrews B. (2016). Predicting swimming performance using state anxiety. *SAJP* 47 110–120. 10.1177/0081246316645060

[B39] Manzoni G. M. Pagnini F. Castelnuovo G. Molinari E. (2008). Relaxation training for anxiety: a ten-years systematic review with meta-analysis. *BMC Psychiatry* 8:41. 10.1186/1471-244X-8-41 18518981 PMC2427027

[B40] Martens R. Burton D. Vealey R. Bump L. Smith D. (1990). “Development and validation of the competitive state anxiety inventory-2 (CSAI-2),” in *Competitive Anxiety in Sport*, eds Martens R. Vealey R. S. Burton D. (Champaign, IL: Human Kinetics), 117–213.

[B41] Modroño C. Guillen F. (2011). Anxiety characteristics of competitive windsurfers: age, gender, performance outcome. *J. Sport Behav.* 34 281–294.

[B42] Munoz A. S. Cayetano A. R. Calle R. C. Blanco F. María J. De Mena Ramos J. M. (2017). Female Spanish athletes face pre-competition anxiety at the highest levels of competition. *Rev. Psicol. Deport.* 26 39–44.

[B43] Neal T. L. Diamond A. B. Goldman S. Klossner D. Morse E. D. Pajak D. E. (2013). Inter-association recommendations for developing a plan to recognize and refer student-athletes with psychological concerns at the collegiate level: an executive summary of a consensus statement. *J. Athl. Train.* 48 716–720. 10.4085/1062-6050-48.4.13 24067154 PMC3784374

[B44] O’Donnell P. S. Dunlap L. L. (2019). Teacher acceptability of progressive muscle relaxation in the classroom for the treatment of test anxiety. *J. Psychol. Couns. Sch.* 29 151–165. 10.1017/jgc.2019.1

[B45] Peirce B. (1852). Criterion for the Rejection of Doubtful Observations. *Astron. J.* 2 161–163. 10.1086/100259

[B46] Petito A. Altamura M. Iuso S. Padalino F. A. Sessa F. D’Andrea G. (2016). The relationship between personality traits, the 5HTT polymorphisms, and the occurrence of anxiety and depressive symptoms in elite athletes. *PLoS One* 11:e0156601. 10.1371/journal.pone.0156601 27257942 PMC4892635

[B47] Qian M. Y. Wu G. C. Zhu R. C. Zhang S. (2000). Development of the revised eysenck personality questionnaire short scale for Chinese (EPQ-RSC). *Acta Psychol. Sin.* 32 317–323.

[B48] Quinones C. Griffiths M. D. (2019). Reducing compulsive Internet use and anxiety symptoms via two brief interventions: a comparison between mindfulness and gradual muscle relaxation. *J. Behav. Addict.* 8 530–536. 10.1556/2006.8.2019.45 31505967 PMC7044623

[B49] Rathschlag M. Memmert D. (2015). Self-generated emotions and their influence on sprint performance:an investigation of happiness and anxiety. *J. Appl. Sport Psychol.* 27 186–199. 10.1080/10413200.2014.974783 23535977

[B50] Reardon C. L. Hainline B. Aron C. M. Baron D. Baum A. L. Bindra A. (2019). Mental health in elite athletes: international olympic committee consensus statement. *Br. J. Sports Med.* 53 667–699. 10.1136/bjsports-2019-100715 31097450

[B51] Rice S. M. Gwyther K. Santesteban-Echarri O. Baron D. Gorczynski P. Gouttebarge V. (2019). Determinants of anxiety in elite athletes: a systematic review and meta-analysis. *Br. J. Sports Med.* 53 722–730. 10.1136/bjsports-2019-100620 31097452 PMC6579501

[B52] Rice S. M. Purcell R. De Silva S. Mawren D. McGorry P. D. Parker A. G. (2016). The mental health of elite athletes: a narrative systematic review. *Sports Med.* 46 1333–1353. 10.1007/s40279-016-0492-2 26896951 PMC4996886

[B53] Rocha V. Osório F. (2018). Associations between competitive anxiety, athlete characteristics and sport context: evidence from a systematic review and meta-analysis. *Arch. Clin. Psychiatry* 45 67–74. 10.1590/0101-60830000000160

[B54] Ross S. M. (2003). Peirce’s criterion for the elimination of suspect experimental data. *J. Eng. Technol.* 20 38–41.

[B55] Rumbold J. L. Fletcher D. Daniels K. (2012). Systematic review of stress management interventions with sport performers. *Sport Exerc. Perform. Psychol.* 1 173–193. 10.1037/a0026628

[B56] Scott-Hamilton J. Schutte N. S. Brown R. F. (2016). Effects of a mindfulness intervention on sports-anxiety, pessimism, and flow in competitive cyclists. *Appl. Psychol. Health Well Being* 8 85–103. 10.1111/aphw.12063 26970111

[B57] Shannon S. Hanna D. Haughey T. Leavey G. McGeown C. Breslin G. (2019). Effects of a mental health intervention in athletes: applying self-determination theory. *Front. Psychol.* 10:1875. 10.3389/fpsyg.2019.01875 31456725 PMC6700360

[B58] Siart B. Nimmerichter A. Vidotto C. Wallner B. (2017). Status, stress and performance in track and field athletes during the european games in baku (Azerbaijan). *Sci. Rep.* 7:6076. 10.1038/s41598-017-06461-z 28729707 PMC5519747

[B59] Silva A. Queiroz S. S. Winckler C. Vital R. Sousa R. A. Fagundes V. (2012). Sleep quality evaluation, chronotype, sleepiness and anxiety of paralympic Brazilian athletes: Beijing 2008 paralympic games. *Br. J. Sports Med.* 46 150–154. 10.1136/bjsm.2010.077016 21173008

[B60] Souza R. A. Beltran O. Zapata D. M. Silva E. Freitas W. Z. Junior R. V. (2019). Heart rate variability, salivary cortisol and competitive state anxiety responses during pre-competition and pre-training moments. *Biol. Sport* 36 39–46. 10.5114/biolsport.2018.78905 30899138 PMC6413577

[B61] Spielberger C. D. Gorssuch R. L. Lushene P. R. Vagg P. R. Jacobs G. A. (1983). *Manual for the State-Trait Anxiety Inventory.* Palo Alto, CA: Consulting Psychologists Press.

[B62] Steinberg F. Doppelmayr M. (2015). A brief note on the relationship between anxiety and performance in scuba diving in adolescents: a field study. *Percept. Mot. Skills* 120 960–970. 10.2466/10.25.PMS.120v16x6 26029967

[B63] Tsopani D. Dallas G. Skordilis E. K. (2011). Competitive state anxiety and performance in young female rhythmic gymnasts. *Perceput. Mot. skills* 112 549–560. 10.2466/05.09.20.pms.112.2.549-560 21667763

[B64] Turner P. E. Raglin J. S. (1996). Variability in precompetition anxiety and performance in college track and field athletes. *Med. Sci. Sports Exerc.* 28 378–385. 10.1097/00005768-199603000-00014 8776227

[B65] Wachelka D. Katz R. C. (1999). Reducing test anxiety and improving academic self-esteem in high school and college students with learning disabilities. *J. Behav. Ther. Exp. Psychiatry* 30 191–198. 10.1016/s0005-7916(99)00024-5 10619543

[B66] Wang C. Bannuru R. Ramel J. Kupelnick B. Scott T. Schmid C. H. (2010). Tai chi on psychological well-being: systematic review and meta-analysis. *BMC Complement. Altern. Med.* 10:23. 10.1186/1472-6882-10-23 20492638 PMC2893078

[B67] Wang X. D. Wang X. L. Ma H. (1999). *Rating Scales for Mental Health.* Beijing: China Mental Health Periodical Office.

[B68] Wilson G. S. Raglin J. S. Harger G. J. (2000). A comparison of the STAI and CSAI-2 in five-day recalls of precompetition anxiety in collegiate track and field athletes. *Scand. J. Med. Sci. Sports* 10 51–54. 10.1034/j.1600-0838.2000.010001051.x 10693614

[B69] Wolpe J. (1958). *Psychotherapy by Reciprocal Inhibition.* Stanford: Stanford University Press.

[B70] Woodman T. Hardy L. (2003). The relative impact of cognitive anxiety and self-confidence upon sport performance: a meta-analysis. *J. Sports Sci.* 21 443–457. 10.1080/0264041031000101809 12846532

[B71] Xu S. S. Zhao X. Zhan G. H. Zhang J. (2017). An intervention study of mindfulness-acceptance-commitment on sport performance and psychological benefits among college students. *J. Tianjin Univ. Sport* 32 501–505.

[B72] Yang J. Park K. Shin M. (2019). Effects of ego-depletion and state anxiety on performance changes in dart-throwing tasks:a latent curve model approach reporting trail data for human participants. *Front. Psychol.* 10:2027. 10.3389/fpsyg.2019.02027 31555188 PMC6742915

[B73] Yang J. Peek-Asa C. Corlette J. D. Cheng G. Foster D. T. Albright J. (2007). Prevalence of and risk factors associated with symptoms of depression in competitive collegiate student athletes. *Clin. J. Sport Med.* 17 481–487. 10.1097/JSM.0b013e31815aed6b 17993792

[B74] Zenebe M. D. Gebru T. H. Mulate Z. T. (2016). Relationship between competitive anxiety, confidence and performance among female volleyball players. *Int. J. Phys. Educ. Sports Health* 3 337–342.

[B75] Zhao F. Y. Duan Y. R. Yan H. X. Li A. Hu Y. Zhang Z. (2016). Evaluation of therapy effects of moxibustion combined with Tai Chi and Jacobson’s progressive relaxation training on exercise-induced insomnia. *J. Shenyang Sport Univ.* 35 75–80.

[B76] Zhu B. L. (1994). The revised Chinese norm of the competitive state anxiety inventory (CSAI-2). *J. Psychol. Sci.* 17:385.

